# Surgical treatment of adult right-sided giant Bochdalek hernia

**DOI:** 10.1093/jscr/rjaf027

**Published:** 2025-03-28

**Authors:** Zuhuan Yao, Ai Huang, Li Wan, Shaobo Hu, Jun Nie, Ke Jiang

**Affiliations:** Department of Thoracic Surgery, Union Hospital, Tongji Medical College, Huazhong University of Science and Technology, 1277 Jiefang Avenue, Wuhan 430022, China; Department of Thoracic Surgery, Union Hospital, Tongji Medical College, Huazhong University of Science and Technology, 1277 Jiefang Avenue, Wuhan 430022, China; Department of Thoracic Surgery, Union Hospital, Tongji Medical College, Huazhong University of Science and Technology, 1277 Jiefang Avenue, Wuhan 430022, China; Liver Transplant Center, Union Hospital, Tongji Medical College, Huazhong University of Science and Technology, 1277 Jiefang Avenue, Wuhan 430022, China; Department of Thoracic Surgery, Union Hospital, Tongji Medical College, Huazhong University of Science and Technology, 1277 Jiefang Avenue, Wuhan 430022, China; Department of Thoracic Surgery, Union Hospital, Tongji Medical College, Huazhong University of Science and Technology, 1277 Jiefang Avenue, Wuhan 430022, China

**Keywords:** Bochdalek hernia, adult diaphragmatic hernia, right-sided diaphragmatic hernia, surgical treatment

## Abstract

Bochdalek hernia is a congenital diaphragmatic hernia typically arising from developmental defects in the diaphragm, allowing abdominal organs to protrude into the thoracic cavity. It primarily affects neonates, with adult cases being rare. This report presents a 19-year-old male with a right-sided Bochdalek hernia containing the liver, gallbladder, and colon, causing compression of the right lung and mediastinum, resulting in respiratory distress. CT imaging illustrated the extent of the herniated organs and their impact on thoracic structures. A combined abdominal and thoracic approach was utilized, including adhesiolysis, reduction of herniated organs, and repair of the diaphragmatic defect with a composite mesh. Postoperatively, the patient showed significant respiratory improvement and experienced a favorable recovery. This case underscores the need to consider Bochdalek hernia in adults with unexplained respiratory distress and abnormal imaging findings.

## Introduction

Incomplete closure of the pleuroperitoneal folds, leading to a posterolateral defect in the diaphragm. In 80%–85% of cases, the hernia appears on the left side, with 10%–15% on the right, and bilateral hernias are extremely rare (<2%) [1]. This condition often allows abdominal organs to intrude into the thoracic cavity, causing pulmonary hypoplasia and pulmonary hypertension, frequently leading to severe respiratory distress in newborns. Right-sided Bochdalek hernias containing the colonic flexure are exceedingly rare in adults [2]. This report presents the case of a 19-year-old asymptomatic male diagnosed with Bochdalek hernia during a routine health examination, with herniation of the liver and right colonic flexure into the right thoracic cavity, compressing the right lung. Although prolonged lung compression resulted in significant pulmonary function impairment, the patient had adapted to this state, experiencing no significant discomfort. Following discovery of the right diaphragmatic defect during physical examination, the patient underwent thoracoabdominal surgery with mesh repair. In adults, untreated diaphragmatic hernia can lead to lung compression, respiratory distress, and the potential for intestinal obstruction due to intrusion of abdominal organs into the thoracic cavity. Untreated BH also poses risks of hernia incarceration and strangulation, highlighting the recommendation for early surgical intervention in adult BH cases, barring significant surgical contraindications.

## Case information

**Figure 1:**
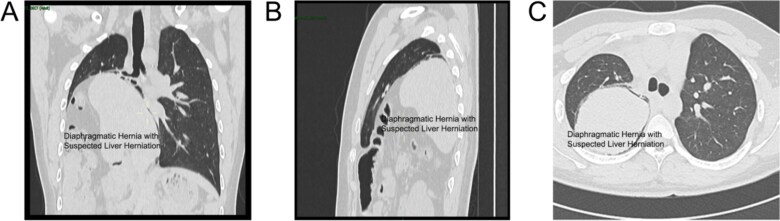
CT scan views of the thoracic hernia with suspected liver tissue involvement: (A) Axial view. (B) Frontal view. (C) Sagittal view.

The patient was a 19-year-old male who experienced shortness of breath following physical activity 3 months earlier. Further examination identified a diaphragmatic hernia, and on 8 July 2024, he sought treatment in the Department of Thoracic Surgery at a hospital. Physical examination showed dullness to percussion in the right thoracic cavity from the second to sixth intercostal spaces, with an elevated liver dullness border. CT imaging confirmed a right-sided Bochdalek hernia with partial herniation of the liver into the thoracic cavity, compression, and atelectasis of the right lung, and mediastinal shift (Fig. 1). Upper gastrointestinal X-ray with contrast identified a filling defect in the duodenal bulb, mucosal irregularity, and proximal jejunum displacement in the upper right abdomen, suggestive of malrotation. A significantly elevated right hemidiaphragm further indicated the presence of a diaphragmatic hernia.

Pulmonary function tests showed severe mixed ventilatory dysfunction (forced vital capacity (FVC) at 45.4% of predicted value and forced expiratory volume in one second/vital capacity (FEV1/VC) at 73.75% of predicted value). ECG revealed sinus arrhythmia with left axis deviation. A multidisciplinary consultation involving hepatobiliary surgery, gastrointestinal surgery, and anesthesiology was conducted to assess treatment options and risks, confirming a diagnosis of right-sided congenital giant diaphragmatic hernia. Preoperative evaluations were completed, and the patient was prepared for surgery under general anesthesia with double-lumen endotracheal intubation.

**Figure 2:**
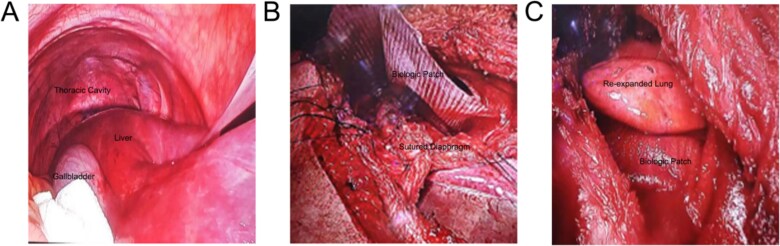
Intraoperative images: (A) Laparoscopic view showing the liver herniated into the right thoracic cavity; (B) Diaphragm repair to secure the mesh; (C) Successful re-expansion of the right lung.

The patient's organs had been in an abnormal position for an extended period, causing significant friction between organs due to breathing and daily activities, leading to severe adhesions. Preoperative CT showed that the liver was almost entirely located within the right thoracic cavity (Fig. 2A), which was further confirmed through advanced laparoscopic exploration during surgery. The surgery began with an abdominal procedure, where adhesiolysis was performed, and the right triangular ligament of the liver was incised to mobilize the liver and prevent it from retracting back into the thoracic cavity. Following this, a thoracic procedure was performed to separate the liver from its adhesions to the pericardium. To repair the diaphragmatic defect, non-absorbable Ethibond sutures were used for closure, and a 15 cm × 20 cm composite mesh was placed on the thoracic side, secured with lateral staples and medial sutures (Fig. 2B). After the diaphragm repair, lung inflation demonstrated satisfactory re-expansion of the right lung (Fig. 2C). An intercostal drainage tube was placed at the surgical site to prevent postoperative pleural effusion. The surgery was completed smoothly, and the patient was transferred to the intensive care unit for further monitoring.

**Figure 3:**
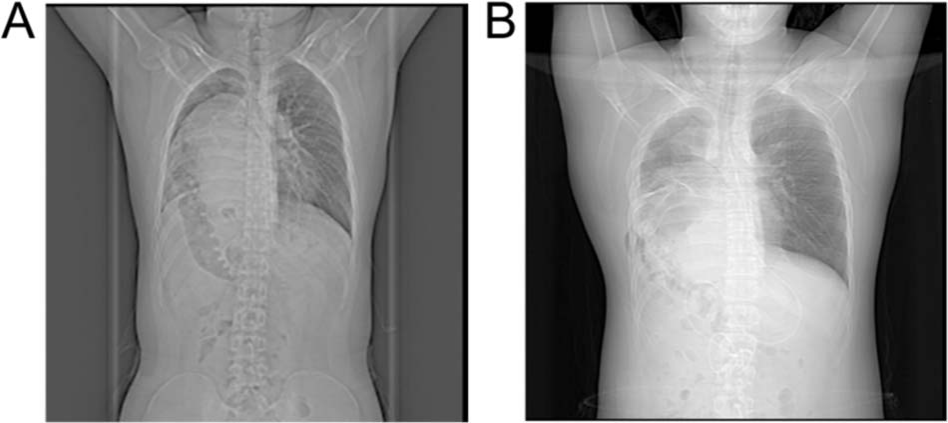
X-ray images: (A) Preoperative X-ray showing a suspected liver mass occupying the right thoracic cavity; (B) Postoperative X-ray showing re-expansion of the right lung and the liver returned to the abdominal cavity.

On postoperative day 3, a follow-up CT scan indicated atelectasis in the right lung due to compression, with displacement of the cardiac silhouette to the left and a small pleural effusion in the right thoracic cavity. The patient participated in lung capacity exercises and chest physiotherapy, gradually recovering respiratory function. Within one week post-surgery, the patient showed significant improvement, with marked relief from dyspnea symptoms. CT imaging demonstrated satisfactory re-expansion of the right lung, and the liver had returned to the abdominal cavity, showing substantial morphological improvement compared to preoperative images (Fig. 3A and B). At a 3-month follow-up, the patient remained asymptomatic, and imaging revealed no recurrence of the hernia or other complications.

## Discussion

Bochdalek hernia is a rare form of congenital diaphragmatic hernia, primarily caused by incomplete development of the diaphragm during the embryonic stage, which allows abdominal organs to herniate into the thoracic cavity. While Bochdalek hernias are most commonly diagnosed in neonates, with an incidence of ~1 in 2500 live births and most cases occurring on the left side, adult right-sided Bochdalek hernias are extremely rare, with an estimated incidence of only 0.17% [3]. Embryologically, diaphragm development begins around the fourth week of gestation and involves fusion of the septum transversum, pleuroperitoneal folds, esophageal mesentery, and abdominal wall musculature. The postero-lateral lumbocostal triangle, a relatively thin area, often becomes an anatomical weak point susceptible to herniation [4].

Symptoms of Bochdalek hernia vary with age and severity. Neonates commonly present with severe respiratory distress, cyanosis, tachypnea, and a flat abdomen due to abdominal organs entering the thoracic cavity and compressing the lungs [5]. Adult Bochdalek hernias may present with mild dyspnea, chest pain, bloating, and nausea, but are frequently asymptomatic, often discovered incidentally during routine imaging [6]. In this case, the patient had a right-sided Bochdalek hernia, with herniation of the liver, gallbladder, and hepatic flexure of the colon into the thoracic cavity, causing significant thoracic space occupation, compression of the right lung, and displacement of the heart and mediastinum, which severely impacted cardiopulmonary function. Due to prolonged abnormal positioning of organs, daily respiration and movement caused severe adhesions between organs, adding complexity to the surgery and necessitating meticulous surgical planning.

The primary treatment for Bochdalek hernia is surgical repair, and in this case, a combined abdominal and thoracic approach was adopted. Typically, Bochdalek hernia repairs involve a midline, paramedian, or subcostal incision. For laparoscopic surgery, the lateral decubitus and reverse Trendelenburg positions are advised, particularly in cases of strangulated hernias where decompression or resection of herniated organs may be required [7]. Diaphragmatic defects are typically closed transversely. Since most Bochdalek hernias lack a hernia sac, caution is warranted when using pneumoperitoneum to prevent intraoperative or postoperative pneumothorax. Synthetic mesh is often used to reinforce the repair and enhance its durability. Across approaches, Bochdalek hernia repairs generally result in good outcomes, with low recurrence rates and potential for improved postoperative pulmonary function [8].

This complex presentation emphasizes the importance of considering Bochdalek hernia in adult patients with unexplained respiratory symptoms and abnormal imaging findings. Additionally, the surgical approach used in this case provides valuable guidance for similar cases.

## Conclusion

Right-sided Bochdalek hernias in adults are rare and often pose diagnostic challenges. This case emphasizes the importance of early detection and multidisciplinary surgical management to prevent severe complications like lung compression and organ strangulation. Successful repair with mesh led to significant respiratory improvement and durable outcomes, highlighting effective treatment strategies.

## Data Availability

Not applicable.
